# Cost-utility of endoscopic screening strategies for upper gastrointestinal cancer across China: a modeling study

**DOI:** 10.3389/fpubh.2025.1643171

**Published:** 2025-08-14

**Authors:** Ruyue Liu, Yifan Li, Xinyi Wang, Yuwang Shang, Nan Zhang, Qiang Sun

**Affiliations:** ^1^Department of Social Medicine and Health Management, School of Public Health, Cheeloo College of Medicine, Shandong University, Jinan, China; ^2^NHC Key Lab of Health Economics and Policy Research (Shandong University), Jinan, China; ^3^Center for Health Management and Policy Research, Shandong University (Shandong Provincial Key New Think Tank), Jinan, China; ^4^Shandong Cancer Hospital and Institute, Shandong First Medical University and Shandong Academy of Medical Sciences, Jinan, China

**Keywords:** cost-utility, upper gastrointestinal cancer, endoscopic screening, comprehensive screening Strategies, Markov model

## Abstract

**Introduction:**

Endoscopic screening for upper gastrointestinal cancer (UGC) is effective, but it's cost-utility across comprehensive strategies remains unclear. We aimed to assess the cost-utility of various endoscopic screening strategies for UGC within the Chinese health care system.

**Methods:**

This study assessed the cost-utility of 40 endoscopic screening strategies using a Markov model. Strategies varied by starting ages (40, 45, 50, or 55 years), screening frequencies (once per lifetime, every 1, 2, 5, 10, or 15 years), and follow-up options. Model parameters were estimated based on our survey data, public surveillance data and published literature. The primary outcome was the incremental cost-utility ratios (ICUR). Deterministic and probabilistic sensitivity analyses were performed to examine key parameters uncertainty.

**Results:**

Seven strategies were identified as the dominant strategies given one-time per capita GDP (¥70,653) of Shandong province in China in 2019. Compared with no screening, all dominant strategies were associated with improved ICUR by CNY ¥12 095.60–31 456.29 per quality-adjusted life years (QALY). Compared with the neighboring strategy, all dominant strategies were associated with improved ICUR by CNY ¥12 095.6266 764.06 per QALY. The y40-nf-il would be the most cost-utility strategy, with probabilities of 42%−95% at 1–3 times the per capita GDP. Findings were robust in all sensitivity analysis.

**Conclusions:**

Comprehensive endoscopic screening strategies for UGC are cost-effective within the Chinese healthcare system. Annual screening starting at age 40 without follow-up emerges as the optimal approach, offering valuable evidence to guide policy development for UGC prevention and control in China.

## 1 Introduction

Globally, upper gastrointestinal cancer (UGC), including esophageal cancer (EC), cardia cancer (CC), and gastric cancer (GC), is one of the highly-fatal malignancies, with an estimated 1.69 million new cases and 1.31 million deaths occurred in 2020 (1). Nearly 50% of those new cases and deaths, respectively, are found in China, and the incidence and mortality rate have increased to the second among all cancers (2, 3). The rising numbers and deaths of UGC confers a substantial burden of suffering on patients and their families, as well as cost to the health care system.

UGC is potentially curable in its early stages. Unfortunately, more than 90% of patients are clinically diagnosed at a late stage, and the survival rate is very poor (4). Endoscopic screening has been proven to be able to identify more early cases of EC, CC, and GC combined, with 5-year survival rate increasing from 20 to 90% (4, 5), and has been widely accepted in some East Asian countries where the incidence rates are high. In Korea, endoscopic screening for GC has been provided to Medicaid participants aged 40 and above once every 2 years since 1999, and currently adjusted to asymptomatic adults aged 40–74 (6). Japan introduced a national screening program in 1983, and recommended endoscopic screening every 2–3 years (7). China has performed endoscopic screening for gastric cancer and esophageal cancer in more than 110 high risk areas throughout the country since 2005 (8). Over the past 20 years, endoscopic screening is always the major secondary prevention measure for UGC.

Some studies have showed that endoscopic screening for EC or GC was cost-effectiveness compared with no screening (9–11). In fact, a single endoscopy examination can completely observe the entire upper digestive tract (esophagus, cardia, and stomach), which can improve screening effectiveness while saving costs (12). However, most studies were conducted on single EC, CC, or GC, and these studies are only trade-off single screening element, such as screening start age, internal among various screening strategies. Only two studies combined EC and GC to develop the endoscopic screening programs. One study (13) analyzed the cost-effectiveness of conducting UGC screening once in the 50-year-old population in the United States. Another study (14) in China fixed screening start and end age, screening interval, and evaluated three different precancerous lesion follow-up periods. In a context of resource constraints, the optimal combination of UGC screening elements including the screening start age, screening interval, and follow-up period remains poorly understood.

Therefore, using the survey data and Markov model, we have estimated the cost-utility of combined endoscopic screening programs for EC, CC, and GC. This study represents the first publication involving the combination of multiple screening elements including screening starting ages, screening interval, and follow-up or not to address a policy question for UGC.

## 2 Materials and methods

### 2.1 Study design

We did an economic evaluation to assess the cost-utility of the various UGC endoscopic screening strategies. The analysis was based on a Markov model constructed by considering the lifetime as time horizon. All data used in our study were collected from the public data sources, published literatures and our cross-sectional surveys. In the survey, we conducted field research on medical institutions and populations including residents and UGC patients in the real world. From the 16 cities located in Shandong Province, five cities (Jinan, Weifang, Jining and Taian, Liaocheng, and Binzhou) were selected using a stratified random sampling, which represent the high, medium and low economic levels, and then six corresponding counties of these five cities were randomly selected for this survey data collection, mainly obtaining the medical costs and health utility values. All participants were asked to provide written informed consent, and the study was approved by the Institutional Ethical Review Board of Shandong Cancer Hospital and Institute.

### 2.2 Model structure

From the health care system perspective, we constructed a transition state Markov decision model using TreeAge Pro (Healthcare Vision) 2021 to evaluate the long-term health outcomes and resource consumption of a hypothetical cohort of 100,000 people undergoing endoscopic screening of UGC, as well as health economic evaluation. The model incorporated 16 health states of disease from a healthy GC/CC/EC to early and advanced cancer (Supplementary material Figure S1) (11, 15–18). It can be seen from the natural history model that patients could transit to another state or remain in the current health state. Generally, before progressing to intramucosal carcinoma, the state can make a bidirectional transition or even regress to a healthy state, but after progressing to intramucosal carcinoma, it can only progress to higher-grade lesions. In the model, the age started at 40 years old and was followed up to 80 years old with a 1-year cycle.

Then, we assumed 40 endoscopic screening strategies that varied in the screening starting ages (40, 45, 50, and 55 years old), screening frequencies (once per lifetime, every year, 2 years, 5 years, 10 and 15 years), and follow-up or not, with no screening as a reference strategy. The 40 strategies were expressed as “y_f/nf_i,” where y represents the screening starting age, f indicates follow up of the population, nf denotes there was no follow up of the population, and i represents screening frequency (Table 1). For example, “y40_nf_i1” indicates a screening strategy starting at age 40, without follow-up, occurring once a year.

**Table 1:** Endoscopic screening strategies for upper gastrointestinal cancer.

**Strategies**	**Strategy code**	**Screening age**	**Screening interval (year)**	**Follow up or not**	**Cumulative number of screening**
1	y40_nf_i1	40–70	1	No	31
2	y40_nf_i2		2	No	16
3	y40_nf_i5		5	No	7
4	y40_nf_i10		10	No	4
5	y40_nf_i15		15	No	3
6	y40_nf_in		Once in a lifetime	No	1
7	y40_f_i5		5	Yes	7
8	y40_f_i10		10	Yes	4
9	y40_f_i15		15	Yes	3
10	y40_f_in		Once in a lifetime	Yes	1
11	y45_nf_i1	45–70	1	No	26
12	y45_nf_i2		2	No	13
13	y45_nf_i5		5	No	6
14	y45_nf_i10		10	No	3
15	y45_nf_i15		15	No	2
16	y45_nf_in		Once in a lifetime	No	1
17	y45_f_i5		5	Yes	6
18	y45_f_i10		10	Yes	3
19	y45_f_i15		15	Yes	2
20	y45_f_in		Once in a lifetime	Yes	1
21	y50_nf_i1	50–70	1	No	21
22	y50_nf_i2		2	No	11
23	y50_nf_i5		5	No	5
24	y50_nf_i10		10	No	3
25	y50_nf_i15		15	No	2
26	y50_nf_in		Once in a lifetime	No	1
27	y50_f_i5		5	Yes	5
28	y50_f_i10		10	Yes	3
29	y50_f_i15		15	Yes	2
30	y50_f_in		Once in a lifetime	Yes	1
31	y55_nf_i1	55–70	1	No	16
32	y55_nf_i2		2	No	8
33	y55_nf_i5		5	No	4
34	y55_nf_i10		10	No	2
35	y55_nf_i15		15	No	2
36	y55_nf_in		Once in a lifetime	No	1
37	y55_f_i5		5	Yes	4
38	y55_f_i10		10	Yes	2
39	y55_f_i15		15	Yes	2
40	y55_f_in		Once in a lifetime	Yes	1

The UGC endoscopic screening strategy consists mainly of screening starting and ending age, screening interval, and follow-up or not. Cancer morbidity and mortality in China are low before age 40 and increase rapidly after age 40. Therefore, 40, 45, 50, and 55 years were used as the screening starting ages. The expert consensus suggests that screening should be avoided in people with a life expectancy <5 years. Based on the population's life expectancy in Shandong Province in 2019 (i.e., 78.94 years), 70 years was set as the screening ending age. In this study, the screening intervals were set at once every year, 2 years, 5 years, 10 years, 15 years, and per lifetime, and only follow-up or not were available.

### 2.3 Model parameters

*Initial distribution probability and Transition probability (TP)* were derived from available public data sources and published literature. The initial distribution probability represents the different upper gastrointestinal diseases or health states of the cohort population when entering the model, which were estimated based on the detection of lesions in the 40–44 age group in the database of “*The Early Diagnosis and Treatment Program of Upper Gastrointestinal Cancer of Shandong province*” from 2015 to 2020. This study assumes that the annual TP between each state is only related to the current state and is a fixed value, with determined from published clinical and epidemiological research (11, 19–27). If the state transition time period in the existing research does not match the time period in the Markov model, a conversion is performed. Details of calculation of the initial distribution probability and TP are shown in Supplementary material Table S1–S3.

*The compliance rates for endoscopic screening and regular follow-up of precancerous lesions* were established at 60% (with a range of 30%−80%) and 75% (with a range of 50%−90%), respectively, according to our survey data and published literature (11, 28, 29). The sensitivity and specificity of endoscopic screening were assumed to be 96% (range from 90 to 99%) and 90% (range from 80 to 95%), respectively, based on the published literature (8, 30–32) (Table 2).

**Table 2:** Parameters used in the model.

**Parameters**	**Base-case value**	**Range^c^**	**Distribution**
Compliance of screening	0.60	0.30–0.80	Triangular (0.30, 0.60, 0.80)
Compliance of follow-up	0.75	0.50–0.90	Triangular (0.50, 0.75, 0.90)
Sensitivity	0.96	0.90–0.99	Triangular (0.90, 0.96, 0.99)
Specificity	0.90	0.80–0.95	Triangular (0.80, 0.90, 0.95)
Discount rate	0.03	0.02–0.05	Triangular (0.02, 0.03, 0.05)
**Utility scores**			
**Esophagus**			
mD	0.944	0.940–1.000	Triangular (0.94, 0.98, 1.00)
MD	0.939	0.930–1.000	Triangular (0.93, 0.98, 1.00)
SD/CIS	0.921	0.881–0.961	β (0.929, 0.145)
Early EC	0.889	0.849–0.929	β (0.904, 0.180)
Advanced EC	0.804	0.764–0.844	β (0.838, 0.223)
**Cardia**			
IM	0.960^a^	0.920–1.000	β (0.972, 0.035)
LGIN	0.941	0.940–1.000	Triangular (0.94, 0.98, 1.00)
HGIN	0.927	0.887–0.967	β (0.936, 0.09)
Early CC	0.863	0.823–0.903	β (0.894, 0.221)
Advanced CC	0.724	0.684–0.764	β (0.757, 0.303)
**Gastric**			
CAG	0.969	0.929–1.000	β (0.972, 0.035)
IM	0.960^a^	0.920–1.000	β (0.972, 0.035)
LGIN	0.930	0.930–1.000	Triangular (0.93, 0.98, 1.00)
HGIN	0.922	0.882–0.962	β (0.937, 0.150)
Early GC	0.828	0.788–0.868	β (0.939, 0.139)
Advanced GC	0.773	0.733–0.803	β (0.803, 0.286)
**Costs**, ¥			
Direct medical cost	629.70	503.76–755.64	
Direct non-medical cost	13.59	10.87–16.31	
Indirect cost	7.97	6.38–9.56	
Total costs	651.36	521.09–781.63	γ (2.16, 0.003)
**Treatment costs**			
**Esophagus**			
SD/CIS	21,950.52	17,560.42–26,340.62	γ (20.43, 0.001)
Early EC	44,891.14	35,912.91–53,869.37	γ (12.23, 0.0003)
Advanced EC (no screening)	76,138.39	60,910.71–91,366.07	γ (26.85, 0.0004)
Advanced EC (screening)	45,683.03^b^	36,546.43–54,819.64	γ (17.13, 0.0004)
**Cardia**			
SD/CIS	25,568.31	20,454.65–30,681.97	γ (4.15, 0.0002)
Early CC	35,162.11	28,129.69–42,194.53	γ (11.04, 0.0004)
Advanced CC (no screening)	64,760.92	51,808.74–77,713.10	γ (22.30, 0.0004)
Advanced CC (screening)	38,856.55^b^	31,085.24–46,627.86	γ (8.10, 0.0002)
SD/CIS	26,891.98	21,513.58–32,270.38	γ (5.45, 0.0002)
Early CC	44,221.23	35,376.98–53,065.48	γ (10.13, 0.0002)
Advanced CC (no screening)	67,278.45	53,822.76–80,734.14	γ (23.85, 0.0004)
Advanced CC (screening)	40,367.07^b^	32,293.66–48,440.48	γ (4.41, 0.0001)
Total costs	651.36	521.09–781.63	γ (2.16, 0.003)

mD, mild atypical hyperplasia; MD, moderate atypical hyperplasia; SD/CIS, severe atypical hyperplasia/carcinoma in situ; IM, intestinal metaplasia; LGIN, low-grade gastric intraepithelial neoplasia; HGIN, high-grade gastric intraepithelial neoplasia; CAG, chronic atrophic gastritis; EC, esophageal cancer; CC, cardia cancer; GC, gastric cancer.

^a^Data were obtained though literature review.

^b^Estimated value (treatment costs of patients with screening findings are about 60% of patients with clinical findings).

^c^Sensitivity analysis range is base-case value ±20%.

*Three types of mortality rates, namely the mortality rate of UGC, the mortality rate excluding UGC, and the annual mortality probability of mid-to-late stage UGC*, are introduced into the model. The data is sourced from the “China Health and Health Statistics Yearbook (2020 Volume).” The mortality of UGC and mortality excluding UGC are introduced into the model as time-dependent variables, which increase with the rise in an individual's age. The annual mortality probability of mid-to-late stage UGC, introduced into the model as a duration-dependent indicator, is positively correlated with the duration of the patient's illness. The mortality probability within the first 5 years is calculated based on the 5-year survival rate of the corresponding cancer. Beyond 5 years, the mortality probability is a constant value, equivalent to the mortality probability in the first-year post-diagnosis (4) (Supplementary material Tables S4,S5).

*Costs* were converted from Chinese CNY to US (US $1 = CNY ¥6.897 in 2019). All costs related to the UGC screening, diagnosis and treatment, including direct medical costs, direct non-medical costs, and indirect costs (33), were calculated from our survey data collected from five hospital institutions, 171 residents, 1,117 patients at different stages of disease (Supplementary material Table S6–S8). Screening, implemented as a public health measure targeting a large population, engages numerous participants and requires substantial resources. Given this, we used 1-time per capita GDP (¥70,653 per QALY) in 2019 as the threshold standard to judge whether a screening strategy is cost-utility. The Willingness to Pay (WTP) was considered to assess the economic feasibility of the screening strategy, providing a measure of how much individuals are willing to spend to gain health benefits.

To calculate quality-adjusted life years (QALY), we further used the European Five-Dimensional Health Scale (EuroQoL-5 Dimensions, EQ-5D-5L) to obtain the various health state utilities of the above 1,117 patients and adopted the Chinese value sets to convert these utilities to the certain utilities applicable to the Chinese population (Supplementary material Table S9). Health utility values ranged from −1 to 1, where 0 represented death and 1 denotes good health (34, 35). A negative utility value was used for a state of health worse than death. These costs and utilities are discounted at a 3% real annual rate (Table 2).

*The primary health outcome* of this study was the incremental cost-utility ratio (ICUR) between the 40 screening strategies. The ICUR was the difference in cost between the 40 strategies divided by the difference in QALYs per patient between the same 40 strategies. When one strategy was more expensive but generated fewer QALYs per patient than a competing strategy, it was dominated by the competing strategy. On the other hand, when one strategy was less expensive but generated more QALYs per patient than a competing strategy, it dominated the competing strategy.

Validation of the final model was divided into three stages: apparent validation, internal validation, and external validation (Supplementary Table S10).

### 2.4 Statistical analysis

#### 2.4.1 Cost-utility analysis

The cost-utility of the screening strategies at different screening starting ages, screening intervals, and follow-up or not were reported in incremental cost-utility ratios (ICURs), defined as incremental costs per QALY gained of each screening strategy compared with no screening or neighboring dominant strategy. We conducted two separate incremental analyses: the main analysis comparing the ICUR of all screening strategies with no screening, and a secondary analysis calculating the ICUR of all screening strategies compared with the neighboring dominant strategy to identify the optimal strategy. High cost-utility was defined as an ICUR less than one-fold the per capita GDP in China. The per capita GDP in China in 2019 was ¥70,653 (36).

#### 2.4.2 Sensitivity analysis

Deterministic sensitivity analyses/Univariate sensitivity analyses were performed to evaluate the effect of all defined variables, and presented it by plotting a tornado diagram. We then used Monte Carlo simulation to perform probability sensitivity analysis, and plot the cost-effectiveness (utility) acceptability curve after 1,000 times of Monte Carlo sampling to evaluate the probability that each screening strategy has cost-effectiveness. Threshold values of CNY¥70,653 per QALY saved are used to identify the optimal strategy for different areas of economic development.

## 3 Results

### 3.1 Cohort population

The study included a hypothetical sample of 100,000 individuals aged 40–80 years. Without screening, the model generates 7,945 new cases and 5,613 deaths of UGC from 2019 to 2059, and estimates the cost of treating patients for UGC without screening over this period at 23,715.23 million. All endoscopic screening strategies could reduce UGC incidence (with range of 165–5,417) and mortality (with range of 170–4,042) compared with no screening, corresponding saved QALYs.

### 3.2 Various UGC endoscopic screening strategies

**Figure 1:**
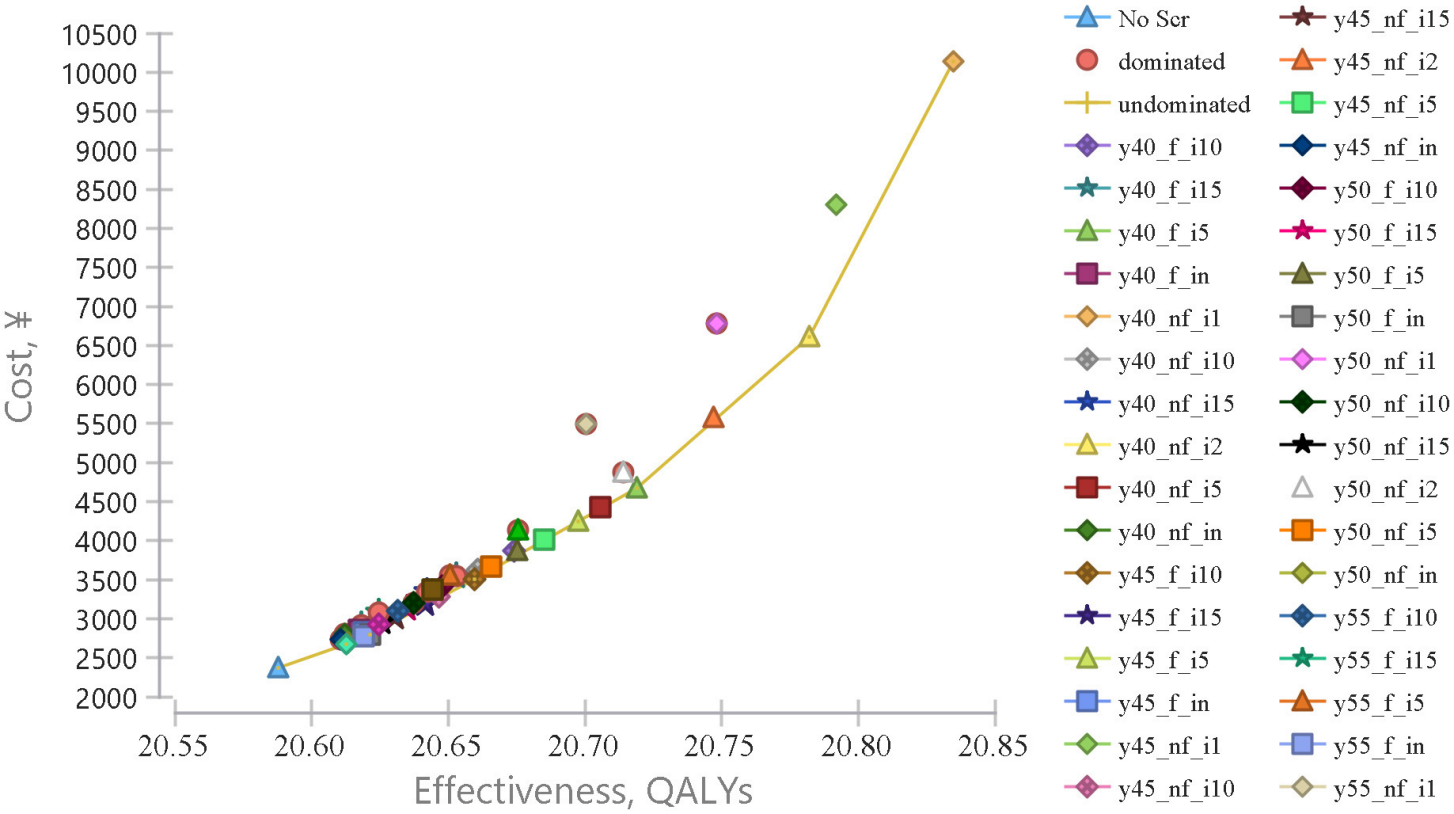
Cost-effectiveness analysis comparing endoscopic screening strategies for upper gastrointestinal cancer. Cost-effectiveness analysis comparing 40 different UGC endoscopic screening strategies vs. no screening, from the age of 40–80 years old. The *x*-axis represents the effectiveness in quality-adjusted life years (QALY) and the *y*-axis represents the cost in RMB (¥).

The cost utility analysis of 40 potential screening strategies identified 7 dominant strategies including y55**_**nf**_**in, y50**_**f**_**in, y45**_**f**_**i10, y45**_**f**_**i5, y40**_**f**_**i5, y40**_**nf**_**i2, and y40**_**nf**_**i1 (Figure 1). Compared with no screening, all seven dominant strategies increased incremental QALYs and costs by 2,486.11–24,705.83 QALYs and CNY ¥30–78 million, and the corresponding ICURs were CNY ¥12,095.60–31,456 per QALY, which were lower than the 1-time per capita GDP (¥70,653). Obviously, all dominant screening strategies would be more cost-utility than no screening (Table 3).

**Table 3:** Cost-utility of dominance strategies vs. no screening and neighboring strategy.

**Strategies**	**Cost (¥, 10K)**	**QALYs**	**CUR (¥/QALY)**	**Incremental analysis (compared with no screening)**			**Incremental analysis (compared with neighboring strategy)**		
				**Incremental cost (**¥**, 10K)**	**Incremental QALYs**	**ICUR (**¥**/QALY)**	**Incremental cost (**¥**, 10K)**	**Incremental QALYs**	**ICUR (**¥**/QALY)**
**Strategies**									
No Scr	23,715.23	2,058,777.70	115.19	–	–	–	–	–	–
y55_nf_in	26,722.33	2,061,263.81	129.64	3,007.10	2,486.11	12,095.60	3,007.10	2,486.11	12,095.62
y50_f_in	27,933.22	2,062,142.13	135.46	4,217.99	3,364.43	12,537.01	1,210.90	878.32	13,786.54
y45_f_i10	35,091.79	2,065,953.84	169.86	11,376.56	7,176.14	15,853.31	7,158.57	3,811.71	18,780.47
y45_f_i5	42,462.37	2,069,733.62	205.16	18,747.14	10,955.92	17,111.42	7,370.57	3,779.78	19,500.01
y40_f_i5	46,736.51	2,071,877.61	225.58	23,021.28	13,099.91	17,573.62	4,274.15	2,143.99	19,935.48
y40_nf_i2	66,070.35	2,078,187.23	317.92	42,355.12	19,409.53	21,821.82	19,333.84	6,309.63	30,641.81
y40_nf_i1	101,430.60	2,083,483.53	486.83	77,715.37	24,705.83	31,456.29	35,360.25	5,296.30	66,764.06

QALY, quality-adjusted life year; CUR, cost-utility ratio; ICUR, incremental cost-utility ratio.

Further comparisons of the neighboring dominant strategies were performed (Table 3). When a dominant strategy was compared with the next most effective screening strategy, 2,486.11–5,296.30 QALYs would be gained and an additional cost of CNY ¥3,007.10 to ¥35,360.25, and the corresponding ICURs were CNY ¥12,095.62–66,764.06 per QALY. The younger the initial screening age would be more cost-utility than the older the initial screening age, and the high-frequency screening strategy would be more cost-utility than the low-frequency screening strategy. It would be cost-utility with or without follow-up. Therefore, y40_nf_i1 would be the optimal strategy, with the ICUR of CNY ¥66,764.06/QALY.

### 3.3 Sensitivity analysis

**Figure 2:**
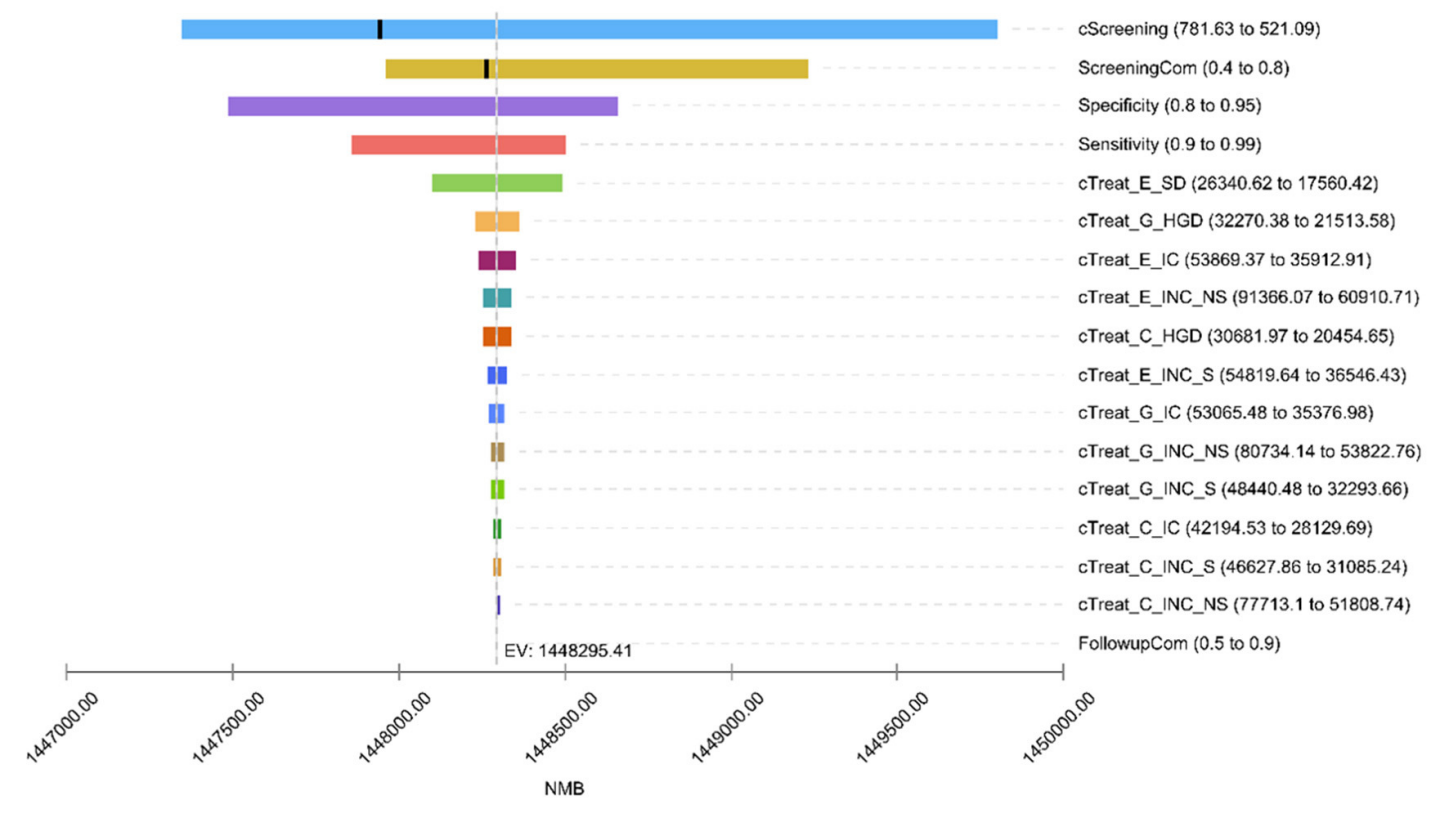
One-way sensitivity analysis tornado diagram. Due to the large impact of the discount rate (DR) on net monetary returns, to ensure the visibility of other factors, the DR Is not shown in the hurricane chart impact. cScreening, screening cost; ScreeningCom, screening compliance; cTreat, treatment cost; E, esophageal cancer; G, gastric cancer; C, cardia cancer; SD, severe atypical hyperplasia/carcinoma *in situ*; HGD, high-grade gastric intraepithelial neoplasia; IC, early-stage cancer; INC, advanced cancer; S, cases detected by screening; NS, cases detected by clinical; FollowupCom, follow up compliance. Since the DR affected the net monetary benefit greatly, the effect of DR is not shown in the tornado diagram (TD) to ensure visibility of other factors.

The one-way sensitivity analysis revealed that the top 6 factors with the largest effects on the model results were discount rate (DR), screening cost (cScreening), screening compliance (ScreeningCom), specificity, sensitivity, and SD/CIS treatment cost (cTreat_E_SD; Figure 2). Increasing the value of DR (>0.0333), cScreening (>682.09) and ScreeningCom (>0.6199), the optimal strategy would change from y40_nf_i1 to y40_nf_i2. Conversely, regardless of the values for sensitivity, specificity, or treatment cost, the optimal strategy remained y40_nf_i1, indicating that these parameters exert a minimal impact on the decision-making process (Supplementary material Figure S2).

**Figure 3:**
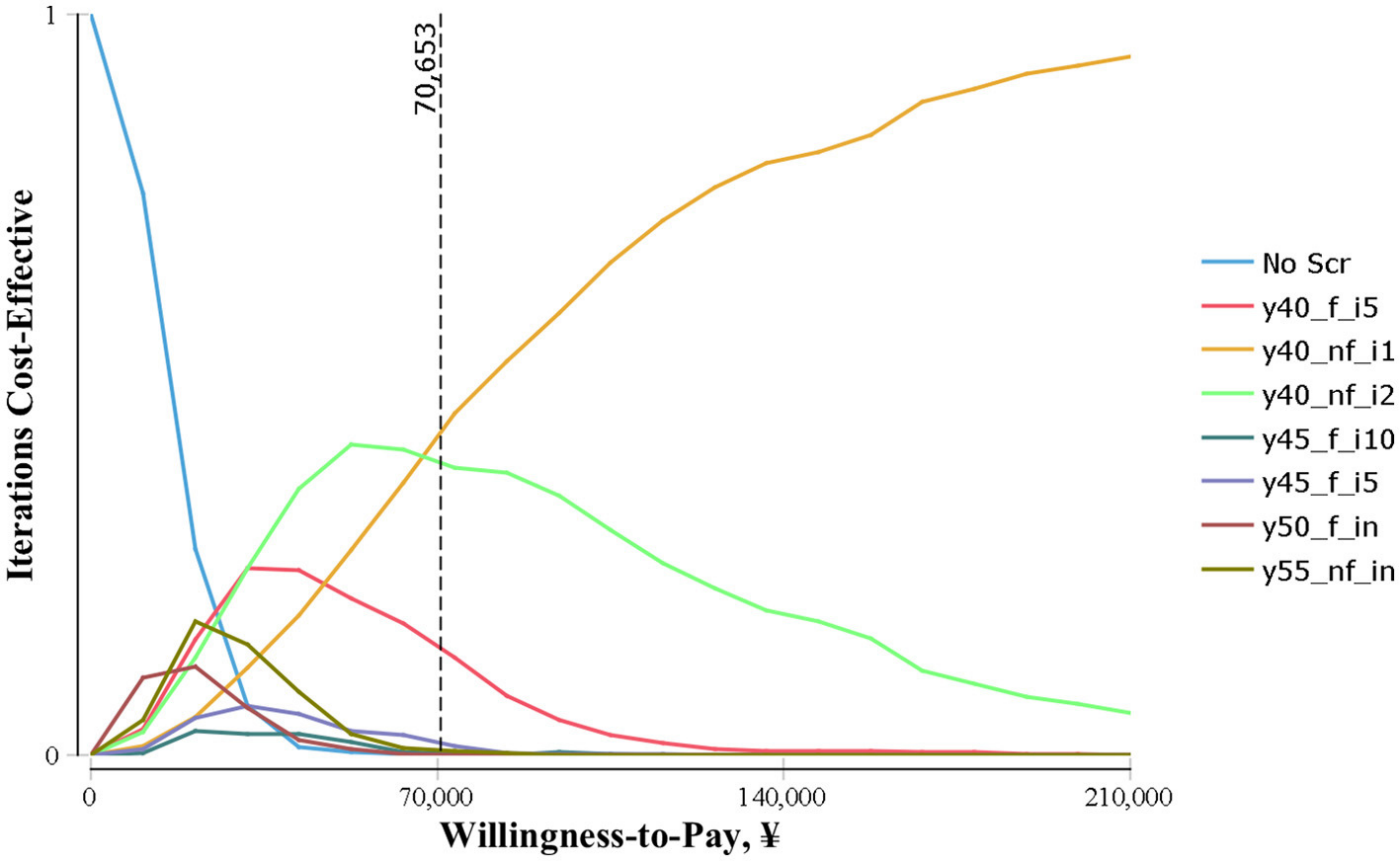
Cost-effectiveness acceptability curve of endoscopic screening strategies for UGC.

The results of the probabilistic sensitivity analyses conducted for each strategy compared with all other strategies are shown in Figure 3. As a result, at a WTP lower than CNY ¥70,653/QALY, the probability of the y40_nf_i2 being cost-effective is highest (25%−45%). When the WTP was CNY ¥70,000/QALY and above, the probability of y-40-il being the optimal strategy was the highest.

## 4 Discussion

Endoscopic screening has been proven to be highly effective in reducing UGC morbidity and mortality (8, 37–39), and its effective were impacted by various factors. However, there were little evidence about the cost-utility of endoscopic screening strategies combined multiple factors. To our knowledge, our study is the first to estimate the cost-utility of UGC endoscopic screening strategies combined of multiple screening elements including screening starting ages, screening interval, and follow-up or not. Our findings revealed that comprehensive endoscopic screening for UGC is cost-utility, and seven dominant strategies were selected.

The cost-utility analysis of the dominant strategies found that the cost per LY gained and per QALY gained gradually increases with the screening effect improving. Compared with no screening, the ICURs of all dominant strategies were lower than CNY ¥40,000/QALY. Compared with the neighboring dominant strategies, the ICURs of all dominant strategies were also lower than CNY ¥40,000/QALY, except for the optimal strategy (y40_nf_i1), with an ICUR of CNY ¥66,764.06/QALY. Therefore, at the level of per capita GDP of CNY ¥70,653 in Shandong Province in 2019, all dominant strategies are economically feasible. However, caution should be taken to the gaps in technology, economic, and resources in different regions of China. In economically underdeveloped areas (per capita GDP lower than CNY ¥70,653), the y40_nf_i2 strategy is the most cost-effective; in economically developed areas (per capita GDP exceeding CNY ¥70,653), y40_nf_il is the most economical. Xia et al (10) conducting study of upper digestive tract cancer screening also showed that the screening effect is closely related to the screening interval. The cost per QALY gained increases with the improvement of the effect. The ICUR of all five strategies were significantly lower than China's per capita GDP ($10,276) in 2019. Screening once every 2 years is the most cost-effective scheme in all age groups, but a screening interval of once a year has not been set. Another study (14) also found that the strategy of starting screening at the age of 40 and conducting it once a year has the best effect and is economically feasible under the threshold of 1 time the per capita GDP. Korean scholars (40) conducted an empirical analysis of the current gastric cancer screening program, which screens people over 40 years old every 2 years. They found that the average cost per LY saved is $20,309, which is much lower than the per capita GDP of Korea. A retrospective cohort study in Japan (41) suggested that different screening intervals should be set for different age groups, but this study did not fully consider cost and economic factors. In addition, our study also found that when screening is done once in a lifetime, the effect and economy of starting screening at the ages of 50 and 55 are significantly better than the screening starting at 40 and 45 years old. Although the effectiveness and feasibility of the current endoscopic screening strategy at the national level in our country (starting and ending ages 40–69, once in a lifetime, regular follow-up) have been confirmed (11, 37, 42), consistent with this study, some scholars believe that raising the starting age of lifetime screening to 45, 50, or 55 years old can further reduce costs and improve the effectiveness and efficiency of screening (11, 43, 44).

Currently, the coverage area of UGC screening programs in our country is relatively limited, and the screening strategy is singular. How to determine the screening interval for the population still poses a significant challenge (45). This study believes that, when resources permit, the screening interval should be set as short as possible to achieve greater effects on a good economic basis. If a lifetime interval is still adopted, the starting age should be raised to 50 or 55 years old. On the other hand, our country has a vast territory and large regional differences. Areas with different levels of economic development often face a series of alternative feasible strategies. It is necessary to make a trade-off between effects, costs, and economy based on value judgment. Sometimes, other variables need to be introduced, such as the screening needs and willingness of the target population, to determine the best setting among many alternative strategies. Ultimately, optimizing UGC screening strategies requires a nuanced understanding of regional disparities, resource allocation, and community engagement to ensure equitable health outcomes for all.

Univariate and probabilistic sensitivity analyses revealed that the discount rate, screening cost, and screening compliance could lead to changes in the optimal strategy. When the discount rate exceeded 3.33%, the optimal strategy changes to y40_nf_i2, which is consistent with the results of two breast cancer screening studies (46, 47). When the screening cost exceeded CNY ¥682.09, the optimal screening strategy also changed from y40_nf_il to y40_nf_i2. Once the screening participation rate reaches 61.99%, y40_nf_i1 is no longer the optimal strategy. As the participation rate increases, the economics of strategies with longer screening intervals gradually improves, especially y40_nf_i2 and y45_nf_i2 gradually become the top two economic strategies. It can be seen that the participation rate has a significant impact on the effectiveness and economy of screening. If a high participation rate can be ensured, the screening interval can be appropriately relaxed, which will help control screening costs and improve screening efficiency. In addition, the sensitivity and specificity of endoscopic screening, as well as the treatment costs of various levels of lesions, also have a certain impact on the cost-effectiveness of screening, but they do not affect the choice of the optimal strategy. Xia et al (10) found that the health utility values of lesions at all levels of the upper digestive tract and the screening compliance are the most important sensitivity factors. They pointed out that improving the screening compliance of the target population is key to achieving preventive effects. Wei (43) found that the cost of endoscopic screening and the treatment of mid-to-late-stage esophageal cancer have a significant impact on the cost-utility of screening. American scholars (13) have found that factors such as the cost of endoscopic screening, the health utility values and progression rates of some lesions, and the compliance with screening and follow-up can cause at least a 20% variation in ICER, but they have a smaller impact on the choice of the optimal strategy. Other studies (48) have also found that the age of screening and the incidence rate of the cancer being screened are also important influencing factors.

Screening costs and the participation rate of the target population are sensitivity factors that should be given priority attention and control. If the screening cost can be reduced in various ways, it can greatly improve the cost-effectiveness of schemes with shorter screening intervals. The participation rate is also considered one of the most important and easily improved sensitivity factors in other disease screenings (49, 50). It can be improved through health education, improving the accessibility of screening services, and enhancing the comfort of endoscopic screening. This has a more significant impact on the cost-effectiveness of schemes with longer screening intervals, such as once every 2 years. If a high participation rate can be ensured, it may be appropriate to relax the screening interval.

There are, however, some limitations in the present study. First, the predictive accuracy of the Markov model largely depends on the accuracy of the parameters. However, in this study, some of the model's parameters come from literature and secondary data, which may affect the accuracy of the prediction results. Second, the gaps in the existing literature required us to base some transition probabilities among health states in our Markov model on non-Chinese data, leading to potential inaccuracies. Third, we collected the screening costs and treatment costs of upper digestive tract lesions through a cross-sectional questionnaire survey, which may be subject to recall bias.

## 5 Conclusions

In considering implementation of a screening strategy, decision makers will need to evaluate multiple factors that could affect both the QALYs saved and the cost and cost-utility of the strategies. Our simulations considering the multiple factors including screening starting age, screening internal and follow-up or not to explore the cost-utility of various comprehensive screening strategies. The findings suggested that from the perspective of the health care system, combined endoscopic screening for EC, GC, and CC would be highly cost-utility for population aged 40–80 years in areas of China; screening at the age of 40, conducting it every year and without follow up would be the optimal strategy. These findings provide evidence for policy development aimed at the sustainable prevention and control of upper gastrointestinal cancers in China.

## Data Availability

The original contributions presented in the study are included in the article/Supplementary material, further inquiries can be directed to the corresponding authors.
