# Supplementary material for: Cost-utility of endoscopic screening strategies for upper gastrointestinal cancer across China: a modeling study
Source: Front Public Health. 2025 Aug 14;13:1643171. doi: 10.3389/fpubh.2025.1643171 (PMC12392997; doi:10.3389/fpubh.2025.1643171)
Supplement: Supplementary file 1 [file Data_Sheet_1.docx]

**Supplementary online content**

**eMethods.**

**eFigure 1.** Natural history of upper gastrointestinal cancer (including esophageal cancer, gastric cancer and cardia cancer)

**eTable 1.** Detection rate of upper gastrointestinal lesions by sex and age in the sample area, 2015-2020 (%)

**eTable 2.** Initial distribution probability of upper gastrointestinal lesions

**eTable 3.** Transition probability of upper gastrointestinal lesions

**eTable 4.** Age-specific mortality of China rural residents in 2019（1/105）

**eTable 5.** Mortality of UGC invasive carcinoma（%）

**eTable 6.** Basic Characteristics of Sample Hospitals

**eTable 7.** Total Cost of Upper Gastrointestinal Cancer Screening and Diagnosis

**eTable 8.** Costs of EC/GC/CC-related treatment in different disease progression stages based on the survey included in our project (RMB, ¥)

**eTable 9.** Health Utility Values of Various Levels of Upper Gastrointestinal Lesions

**eTable10.** Comparison of model-predicted life expectancy with real data

**eResults.**

**eFigure 2.** One-way sensitivity analysis of primary factors

**eReference.**

**eMethods.**

**1 Markov model**

**Natural history of upper gastrointestinal cancer**

According to domestic and international studies[1-5], the natural history of upper gastrointestinal cancer (UGC) is shown in **eFigure 1**. It can be seen from the natural history model that patients could transit to another state or remain in the current health state. Generally, before progressing to intramucosal carcinoma, the state can make a bidirectional transition or even regress to a healthy state, but after progressing to intramucosal carcinoma, it can only progress to higher-grade lesions[5].

**eFigure 1. Natural history of upper gastrointestinal cancer (including esophageal cancer, gastric cancer and cardia cancer).**


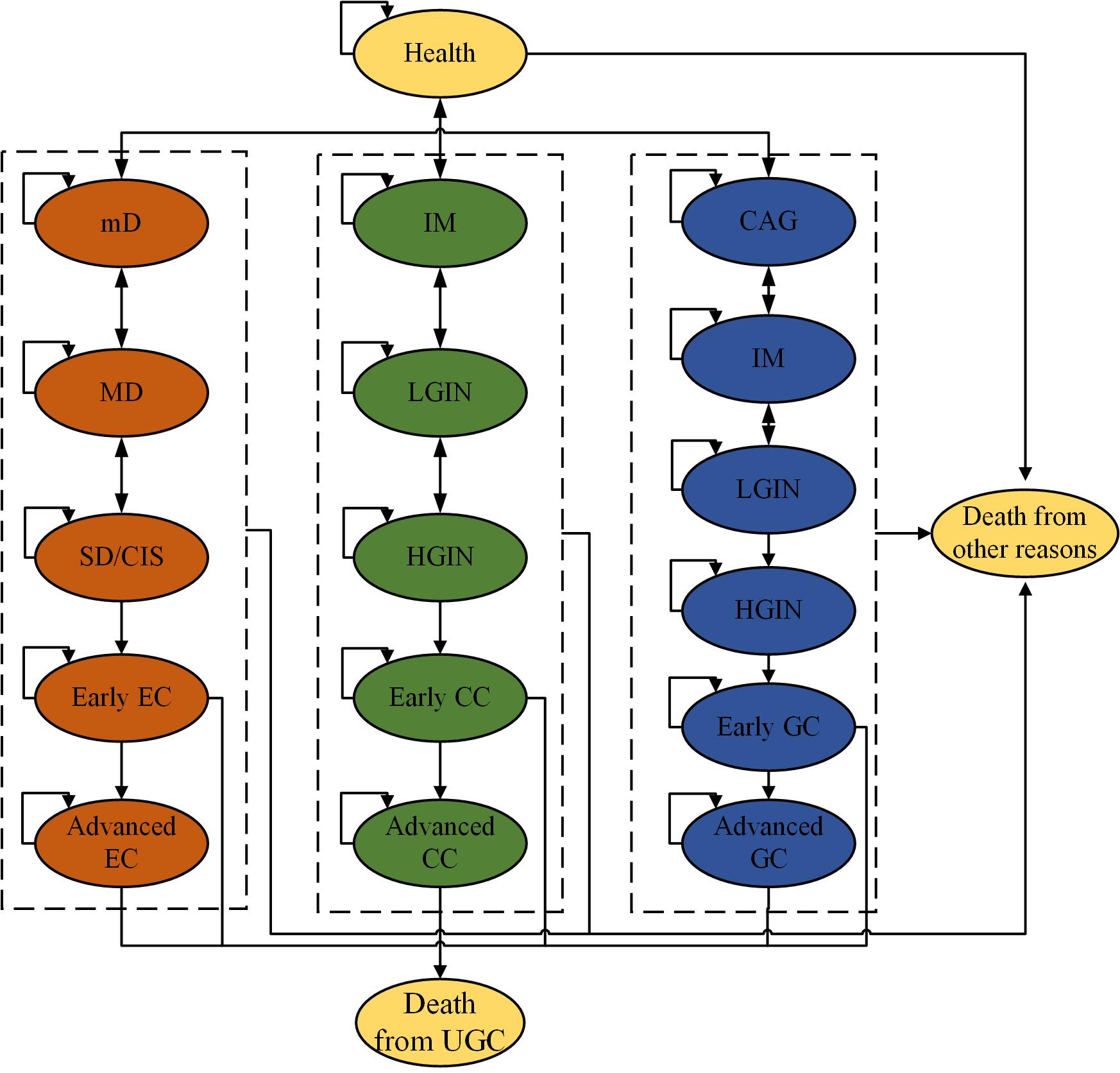


Abbreviations: mD, mild atypical hyperplasia; MD, moderate atypical hyperplasia; SD/CIS, severe atypical hyperplasia/carcinoma in situ; IM, intestinal metaplasia; LGIN, low-grade gastric intraepithelial neoplasia; HGIN, high-grade gastric intraepithelial neoplasia; CAG, chronic atrophic gastritis; EC, esophageal cancer; CC, cardia cancer; GC, gastric cancer; UGC, upper gastrointestinal cancer.

**2 Model parameters**

**2.1 Initial distribution probabilities and transition probabilities**

The initial distribution state is the different disease states that the cohort population was already in before entering circulation. In this study, the initial distribution state of the simulated cohort population was estimated based on the detection of lesions in the 40-44 age group in the database of “*The Early Diagnosis and Treatment Program of Upper Gastrointestinal Cancer of Shandong province*” from 2015 to 2020 in the sample area **(eTable 1)**. The results related initial distributionprobabilities are shown in **eTable 2.**

**eTable 1. Detection rate of upper gastrointestinal lesions by sex and age in the sample area, 2015-2020 (%)**

| **Disease site** | **Lesion** | **Male** | | | | | | | **Female** | | | | | | | **Total** | | | | | | |
| --- | --- | --- | --- | --- | --- | --- | --- | --- | --- | --- | --- | --- | --- | --- | --- | --- | --- | --- | --- | --- | --- | --- |
|  |  | **40-** | **45-** | **50-** | **55-** | **60-** | **65-** | **Total** | **40-** | **45-** | **50-** | **55-** | **60-** | **65-** | **Total** | **40-** | **45-** | **50-** | **55-** | **60-** | **65-** | **Total** |
| Esophagus | Normal | 82.85 | 80.55 | 75.93 | 75.86 | 71.34 | 70.92 | 75.58 | 88.46 | 86.03 | 81.55 | 82.45 | 78.17 | 78.30 | 81.90 | 85.93 | 83.57 | 79.12 | 79.43 | 75.01 | 74.96 | 79.05 |
|  | inflammation | 12.46 | 14.11 | 17.28 | 16.88 | 20.09 | 18.78 | 17.08 | 8.63 | 10.62 | 14.17 | 13.02 | 16.69 | 15.95 | 13.66 | 10.36 | 12.19 | 15.52 | 14.79 | 18.26 | 17.23 | 15.20 |
|  | mD | 2.82 | 3.09 | 4.05 | 4.71 | 5.08 | 5.97 | 4.41 | 1.99 | 2.42 | 2.81 | 3.15 | 3.40 | 3.61 | 2.98 | 2.36 | 2.72 | 3.35 | 3.87 | 4.18 | 4.68 | 3.62 |
|  | MD | 0.78 | 0.75 | 0.86 | 0.80 | 1.23 | 1.33 | 0.96 | 0.50 | 0.56 | 0.81 | 0.53 | 0.65 | 0.71 | 0.65 | 0.63 | 0.65 | 0.84 | 0.65 | 0.92 | 0.99 | 0.79 |
|  | SD/CIS | 0.51 | 0.75 | 0.85 | 0.90 | 1.03 | 1.27 | 0.91 | 0.22 | 0.16 | 0.42 | 0.52 | 0.56 | 0.74 | 0.45 | 0.35 | 0.43 | 0.61 | 0.70 | 0.78 | 0.98 | 0.66 |
|  | intramucosal carcinoma | 0.37 | 0.41 | 0.65 | 0.36 | 0.67 | 0.86 | 0.56 | 0.14 | 0.08 | 0.14 | 0.18 | 0.21 | 0.37 | 0.19 | 0.25 | 0.23 | 0.36 | 0.27 | 0.42 | 0.59 | 0.36 |
|  | submucosal carcinoma | 0.10 | 0.16 | 0.23 | 0.28 | 0.29 | 0.47 | 0.27 | 0.03 | 0.07 | 0.06 | 0.07 | 0.18 | 0.28 | 0.11 | 0.06 | 0.11 | 0.13 | 0.17 | 0.23 | 0.36 | 0.18 |
|  | Invasive Carcinoma | 0.10 | 0.16 | 0.15 | 0.21 | 0.28 | 0.41 | 0.22 | 0.03 | 0.07 | 0.04 | 0.08 | 0.14 | 0.05 | 0.07 | 0.06 | 0.11 | 0.09 | 0.14 | 0.20 | 0.21 | 0.14 |
|  | Total | 100 | 100 | 100 | 100 | 100 | 100 | 100 | 100 | 100 | 100 | 100 | 100 | 100 | 100 | 100 | 100 | 100 | 100 | 100 | 100 | 100 |
| Cardia | Normal | 93.14 | 91.37 | 90.77 | 90.35 | 88.45 | 87.55 | 90.05 | 93.42 | 93.27 | 92.03 | 90.82 | 89.60 | 88.14 | 91.09 | 93.29 | 92.42 | 91.48 | 90.60 | 89.07 | 87.87 | 90.62 |
|  | NAG | 4.79 | 5.31 | 5.35 | 5.54 | 6.19 | 6.46 | 5.66 | 5.01 | 4.46 | 5.35 | 5.80 | 6.05 | 6.18 | 5.52 | 4.91 | 4.84 | 5.35 | 5.68 | 6.11 | 6.31 | 5.58 |
|  | AG | 1.12 | 1.87 | 1.88 | 2.10 | 2.51 | 2.22 | 2.04 | 0.98 | 1.31 | 1.40 | 1.79 | 2.19 | 2.66 | 1.76 | 1.04 | 1.56 | 1.61 | 1.93 | 2.34 | 2.46 | 1.88 |
|  | LGIN | 0.78 | 1.26 | 1.54 | 1.45 | 1.77 | 1.94 | 1.52 | 0.48 | 0.80 | 0.86 | 1.15 | 1.41 | 1.57 | 1.08 | 0.61 | 1.01 | 1.16 | 1.29 | 1.58 | 1.74 | 1.28 |
|  | HGIN | 0.07 | 0.07 | 0.16 | 0.23 | 0.47 | 0.69 | 0.29 | 0.03 | 0.08 | 0.13 | 0.11 | 0.28 | 0.40 | 0.18 | 0.05 | 0.07 | 0.15 | 0.17 | 0.36 | 0.53 | 0.23 |
|  | intramucosal carcinoma | 0.03 | 0.07 | 0.19 | 0.14 | 0.36 | 0.54 | 0.23 | 0.03 | 0.03 | 0.13 | 0.18 | 0.26 | 0.49 | 0.19 | 0.03 | 0.04 | 0.16 | 0.17 | 0.31 | 0.51 | 0.21 |
|  | submucosal carcinoma | 0.03 | 0.05 | 0.06 | 0.14 | 0.23 | 0.41 | 0.16 | 0.03 | 0.03 | 0.08 | 0.08 | 0.11 | 0.43 | 0.12 | 0.03 | 0.04 | 0.07 | 0.11 | 0.17 | 0.42 | 0.14 |
|  | Invasive Carcinoma | 0.03 | 0.02 | 0.05 | 0.05 | 0.03 | 0.19 | 0.06 | 0.03 | 0.03 | 0.02 | 0.07 | 0.09 | 0.12 | 0.06 | 0.03 | 0.02 | 0.03 | 0.06 | 0.06 | 0.15 | 0.06 |
|  | Total | 100 | 100 | 100 | 100 | 100 | 100 | 100 | 100 | 100 | 100 | 100 | 100 | 100 | 100 | 100 | 100 | 100 | 100 | 100 | 100 | 100 |
| Gastric | Normal | 80.07 | 77.01 | 76.71 | 74.00 | 75.00 | 73.31 | 75.64 | 80.99 | 79.18 | 77.26 | 75.68 | 75.94 | 73.33 | 76.74 | 80.57 | 78.21 | 77.02 | 74.91 | 75.51 | 73.32 | 76.24 |
|  | NAG | 13.24 | 14.20 | 13.30 | 14.93 | 13.49 | 13.91 | 13.90 | 12.83 | 13.34 | 14.45 | 14.26 | 13.34 | 14.52 | 13.90 | 13.01 | 13.73 | 13.95 | 14.57 | 13.41 | 14.24 | 13.90 |
|  | AG | 4.89 | 6.12 | 6.78 | 7.71 | 7.23 | 7.17 | 6.86 | 4.56 | 5.26 | 6.14 | 7.17 | 7.29 | 8.18 | 6.59 | 4.71 | 5.65 | 6.42 | 7.42 | 7.26 | 7.72 | 6.71 |
|  | IM | 0.44 | 0.67 | 0.61 | 0.66 | 0.88 | 1.08 | 0.74 | 0.56 | 0.63 | 0.45 | 0.61 | 0.61 | 0.51 | 0.56 | 0.51 | 0.65 | 0.52 | 0.63 | 0.73 | 0.77 | 0.64 |
|  | LGIN | 0.98 | 1.74 | 2.18 | 2.03 | 2.51 | 2.97 | 2.16 | 0.92 | 1.41 | 1.30 | 1.73 | 2.19 | 2.35 | 1.69 | 0.95 | 1.56 | 1.68 | 1.87 | 2.34 | 2.63 | 1.90 |
|  | HGIN | 0.17 | 0.10 | 0.15 | 0.19 | 0.36 | 0.71 | 0.27 | 0.06 | 0.12 | 0.14 | 0.24 | 0.22 | 0.37 | 0.20 | 0.11 | 0.11 | 0.15 | 0.22 | 0.28 | 0.52 | 0.23 |
|  | intramucosal carcinoma | 0.14 | 0.10 | 0.10 | 0.21 | 0.27 | 0.43 | 0.20 | 0.03 | 0.03 | 0.17 | 0.21 | 0.24 | 0.45 | 0.20 | 0.08 | 0.06 | 0.14 | 0.21 | 0.25 | 0.44 | 0.20 |
|  | submucosal carcinoma | 0.03 | 0.03 | 0.09 | 0.22 | 0.16 | 0.26 | 0.14 | 0.03 | 0.01 | 0.06 | 0.08 | 0.09 | 0.15 | 0.07 | 0.03 | 0.02 | 0.07 | 0.14 | 0.12 | 0.20 | 0.10 |
|  | Invasive Carcinoma | 0.03 | 0.03 | 0.08 | 0.05 | 0.11 | 0.17 | 0.08 | 0.03 | 0.01 | 0.03 | 0.03 | 0.08 | 0.14 | 0.05 | 0.03 | 0.02 | 0.05 | 0.04 | 0.09 | 0.15 | 0.06 |
|  | Total | 100 | 100 | 100 | 100 | 100 | 100 | 100 | 100 | 100 | 100 | 100 | 100 | 100 | 100 | 100 | 100 | 100 | 100 | 100 | 100 | 100 |

Abbreviations: mD, mild atypical hyperplasia; MD, moderate atypical hyperplasia; SD/CIS, severe atypical hyperplasia/carcinoma in situ; NAG, non-atrophic gastritis; AG, atrophic gastritis; LGIN, low-grade gastric intraepithelial neoplasia; HGIN, high-grade gastric intraepithelial neoplasia; IM, intestinal metaplasia.

**eTable 2. Initial distribution probability of upper gastrointestinal lesions**

| **Lesion** | **Probability** |
| --- | --- |
| Esophagus |  |
| mD | 0.0236 |
| MD | 0.0063 |
| SD/CIS | 0.0035 |
| early EC | 0.0031 |
| advanced EC | 0.0006 |
| Cardia |  |
| IM | 0.0104 |
| LGIN | 0.0061 |
| HGIN | 0.0005 |
| early CC | 0.0006 |
| advanced CC | 0.0003 |
| Gastric |  |
| CAG | 0.0471 |
| IM | 0.0051 |
| LGIN | 0.0095 |
| HGIN | 0.0011 |
| early GC | 0.0008 |
| advanced GC | 0.0003 |

Abbreviations: mD, mild atypical hyperplasia; MD, moderate atypical hyperplasia; SD/CIS, severe atypical hyperplasia/carcinoma in situ; IM, intestinal metaplasia; LGIN, low-grade gastric intraepithelial neoplasia; HGIN, high-grade gastric intraepithelial neoplasia; CAG, chronic atrophic gastritis; EC, esophageal cancer; CC, cardia cancer; GC, gastric cancer

**2.2 Transition probability**

Transition probability (TP) was determined based on data from related clinical studies and epidemiologic research results [6-19]. 6The relevant literature were identified based on the characteristics of this study. Data were extracted from the articles, and equation 1 and equation 2 were used to calculate the TP of the upper gastrointestinal lesions^24^.

*r=-[ln(1-P_1_)]/t_1_* (Equation 1)

*P_2_=1-exp(-rt_2_)* (Equation 2)

Where r refers to instantaneous incidence rate, P_1_ represents the probability of an event occurring over time, P_2_ denotes the transition probability within one cycle, t_1_ indicates the research duration, and t_2_ represents the cycle (values are 1 year or 12 months).

The annual transition probabilities of lesions at all levels are shown in **eTable 3**.

**eTable 3. Transition probability of upper gastrointestinal lesions**

| **Parameter** | **Value** | **Parameter** | **Value** | **Parameter** | **Value** |
| --- | --- | --- | --- | --- | --- |
| Normal |  | IM |  | IM |  |
| To mD | 0.0240 | To normal | 0.1270 | To severe CAG | 0.0380 |
| To MD | 0.0250 | Remain the same | 0.8560 | Remain the same | 0.9260 |
| To CAG | 0.0170 | To LGIN | 0.0170 | To LGIN | 0.0360 |
| mD |  | LGIN |  | LGIN |  |
| To normal | 0.0500 | To IM | 0.0420 | To severe IM | 0.0490 |
| Remain the same | 0.9000 | Remain the same | 0.9160 | Remain the same | 0.9010 |
| To MD | 0.0500 | To HGIN | 0.0420 | To HGIN | 0.0500 |
| MD |  | HGIN |  | HGIN |  |
| To mD | 0.0800 | To LGIN | 0.0880 | To LGIN | 0.0480 |
| Remain the same | 0.8000 | Remain the same | 0.7530 | Remain the same | 0.7030 |
| To SD/CIS | 0.1200 | To early CC | 0.1590 | To early GC | 0.2490 |
| SD/CIS |  | After LGIN treatment |  | After LGIN treatment |  |
| To MD | 0.1700 | To normal | 0.9830 | To normal | 0.9640 |
| Remain the same | 0.7500 | Relapse | 0.0170 | Relapse | 0.0360 |
| To early EC | 0.0800 | Early CC |  | Early GC |  |
| After SD/CIS treatment |  | Remain the same | 0.8640 | Remain the same | 0.5710 |
| To normal | 0.9950 | To advanced CC | 0.1360 | To advanced GC | 0.4290 |
| Relapse | 0.0050 | After early CC treatment |  | After early GC treatment |  |
| Early EC |  | Remain the same | 0.9300 | Remain the same | 0.9260 |
| Remain the same | 0.8500 | Relapse | 0.0160 | Relapse | 0.0150 |
| To advanced EC | 0.1500 | Relapse and progress to advanced CC | 0.0540 | Relapse and progress to advanced GC | 0.0590 |
| After early EC treatment |  | Advanced CC |  | Advanced GC |  |
| Remain the same | 0.8500 | Stable after treatment | 0.8370 | Stable after treatment | 0.7940 |
| Relapse | 0.0500 | Relapse after treatment | 0.1630 | Relapse after treatment | 0.2060 |
| Relapse and progress to advanced EC | 0.1000 | Severe CAG |  |  |  |
| Advanced EC |  | To normal | 0.0160 |  |  |
| Stable after treatment | 0.7696 | Remain the same | 0.9050 |  |  |
| Relapse after treatment | 0.2304 | To severe IM | 0.0790 |  |  |

Abbreviations: mD, mild atypical hyperplasia; MD, moderate atypical hyperplasia; SD/CIS, severe atypical hyperplasia/carcinoma in situ; IM, intestinal metaplasia; LGIN, low-grade gastric intraepithelial neoplasia; HGIN, high-grade gastric intraepithelial neoplasia; CAG, chronic atrophic gastritis; EC, esophageal cancer; CC, cardia cancer; GC, gastric cancer.

**2.3** Mortality Distribution

We divided the mortality into 3 categories: population mortality, non-UGC mortality, and UGC mortality. Population mortality and non-UGC mortality were calculated based on data from the China Health Statistical Yearbook 2020. The intermediate and advanced UGC mortality was calculated based on a related study by the National Cancer Center^[20]^. The annual death rate for clinically cured patients was used as the annual death rate at the first year of diagnosis because no studies have reported their mortality **(eTable 4 and eTable 5)**.

**eTable 4. Age-specific mortality of China rural residents in 2019（1/10^5^）**

| **Parameters** | **40-44y** | **45-49y** | **50-54y** | **55-59y** | **60-64y** | **65-69y** | **70-74y** | **75-79y** | **80-84y** | **85-** |
| --- | --- | --- | --- | --- | --- | --- | --- | --- | --- | --- |
| Population mortality | 146.37 | 226.76 | 376.42 | 570.55 | 838.69 | 1439.09 | 2660.35 | 4704.96 | 7668.76 | 19718.49 |
| UGC mortality | 4.05 | 9.12 | 19.72 | 35.70 | 63.34 | 107.37 | 183.95 | 262.58 | 297.52 | 401.53 |
| -EC mortality | 0.73 | 2.59 | 6.80 | 13.92 | 25.10 | 43.25 | 73.31 | 103.20 | 122.19 | 161.54 |
| -CC & GC mortality | 3.32 | 6.53 | 12.92 | 21.78 | 38.24 | 64.12 | 110.64 | 159.38 | 175.33 | 239.99 |
| Non-UGC mortality | 142.32 | 217.64 | 356.70 | 534.85 | 775.35 | 1331.72 | 2476.40 | 4442.38 | 7374.24 | 19316.96 |

**eTable 5. Mortality of UGC invasive carcinoma（%）**

| **Parameters** | **Disease retention time (year)** | | | | | | | |
| --- | --- | --- | --- | --- | --- | --- | --- | --- |
|  | **1** | **2** | **3** | **4** | **5** | **6** | **……** | **n** |
| EC mortality | 21.24 | 37.97 | 51.15 | 61.53 | 69.70 | 21.24 | 21.24 | 21.24 |
| CC mortality | 18.89 | 34.22 | 46.64 | 56.72 | 64.90 | 18.89 | 18.89 | 18.89 |
| GC mortality | 18.89 | 34.22 | 46.64 | 56.72 | 64.90 | 18.89 | 18.89 | 18.89 |

**2.4 Costs of screening and EC/GC/CC-related treatment**

**2.4.1 Direct Medical Costs of Endoscopy Screening and Diagnosis**

All costs related to the UGC screening, diagnosis and treatment, including direct medical costs, direct non-medical costs, and indirect costs[30], were calculated from our survey data collected from 5 hospital institutions, 171 residents, 1117 patients at different stages of disease. The basic situation of the sample hospitals is shown in **eTable6**. The average cost of the UGC endoscopy screening stage is ¥559.28 (including anesthesia costs), and the differences between different regions and different types of hospitals have increased, ranging from ¥200.53 to ¥897.16; the costs of equipment and drugs account for a higher proportion, which are 36.19% and 35.28% respectively. The average cost of the pathological diagnosis stage is ¥70.42, of which the equipment cost accounts for the highest proportion, reaching 55.53%; the total direct medical cost of upper gastrointestinal cancer endoscopy screening and diagnosis is ¥629.70.

The total cost of UGC endoscopy screening in the sample area is ¥651.36, of which the direct medical cost accounts for the highest proportion, at 96.67%, and the direct non-medical cost and indirect cost only account for about 3%. In the economic analysis, a sensitivity analysis is conducted with a range of ¥521.09 to ¥781.63**(eTable 7)**.

The direct medical costs, direct non-medical costs, and indirect costs of treating lesions are shown in **eTable 8**.

**eTable 6. Basic Characteristics of Sample Hospitals**

| **Sample Hospitals** | **Cities** | **Number of beds** | **Number of endoscopic examinations in 2018** |
| --- | --- | --- | --- |
| Linqu County People’s Hospital | Weifang | 1032 | 9250 |
| Pingyin County TCM Hospital | Jinan | 606 | 3800 |
| Feicheng City People’s Hospital | Taian | 1466 | 16000 |
| Liaocheng City Tumor Hospital | Liaocheng | 700 | 2800 |
| Provincial Center for Prevention and Treatment of Digestive System Diseases | Jining | 137 | 4000 |

**eTable 7.** **Total Cost of Upper Gastrointestinal Cancer Screening and Diagnosis**

| **Cost Type** | **Base value(¥)** | **Proportion(%)** | **Range of sensitivity analysis(¥)^a^** | |
| --- | --- | --- | --- | --- |
|  |  |  | **Upper** | **Lower** |
| Direct medical costs | 629.70 | 96.67 | 503.76 | 755.64 |
| Direct non-medical costs | 13.59 | 2.09 | 10.87 | 16.31 |
| Indirect costs | 7.97 | 1.22 | 6.38 | 9.56 |
| Total | 651.36 | - | 521.09 | 781.63 |

Note：^a^ The range of sensitivity analysis is ±20% of the total value；

**eTable 8. Costs of EC/GC/CC-related treatment in different disease progression stages based on the survey included in our project (RMB,** ¥**)**

|  | **Direct medical costs** | **Direct** non-medical **costs** | **Indirect costs** | **Total** | **Range of sensitivity analysis^a^** | | **Distribution** |
| --- | --- | --- | --- | --- | --- | --- | --- |
| **Disease progression stages** |  |  |  |  | **Upper** | **Lower** |  |
| SD/CIS | 19518.00 | 1170.70 | 1261.82 | 21950.52 | 17560.42 | 26340.62 | γ（35.96,0.001） |
| Early EC | 40601.00 | 2398.14 | 1892.00 | 44891.14 | 35912.91 | 53869.37 | γ（78.96,0.002） |
| Invasive EC (clinical) | 65113.00 | 6751.96 | 4273.43 | 76138.39 | 60910.71 | 91366.07 | γ（102.01,0.002） |
| Invasive EC (screening) | - | - | - | 45683.03^b^ | 36546.43 | 54819.64 | γ（26.75,0.0009） |
| LGIN | 22869.00 | 1280.68 | 1418.63 | 25568.31 | 20454.65 | 30681.97 | γ（14.50,0.0006） |
| Early CC | 31671.00 | 1752.52 | 1738.59 | 35162.11 | 28129.69 | 42194.53 | γ（27.34，0.001） |
| Invasive CC (clinical) | 57102.74 | 4747.87 | 2910.31 | 64760.92 | 51808.74 | 77713.10 | γ（82.76,0.002） |
| Invasive CC (screening) | - | - | - | 38856.55^b^ | 31085.24 | 46627.86 | γ（28.95,0.0009） |
| LGIN | 23917.86 | 1686.00 | 1288.12 | 26891.98 | 21513.58 | 32270.38 | γ（40.22,0.001） |
| Early GC | 40913.56 | 1738.07 | 1569.60 | 44221.23 | 35376.98 | 53065.48 | γ（36.91,0.001） |
| Invasive GC (clinical) | 59740.76 | 4345.89 | 3191.80 | 67278.45 | 53822.76 | 80734.14 | γ（106.77,0.002） |
| Invasive GC (screening) | - | - | - | 40367.07^b^ | 32293.66 | 48440.48 | γ（34.52,0.001） |

Note：^a^ The range of sensitivity analysis is ±20% of the total value；^b^ Estimated value (the cost of diagnosing and treating patients found through screening is about 60% of the cost for patients found clinically).

**2.5 Utility scores of EC/GC-related health states**

A survey was conducted using the EQ-5D-5L scale, and after converting the scores using the Chinese utility value scoring system, the health utility values of various levels of upper gastrointestinal lesions were obtained, as shown in **eTable10**. Overall, the health utility value decreases as the severity of the disease increases. The utility values of precancerous lesions are all above 0.9; the utility values of early esophageal, cardia, and gastric cancers are around 0.85, and the utility values of middle and late-stage cancers are the lowest, which are 0.804, 0.724, and 0.773 respectively.

**eTable 9. Health Utility Values of Various Levels of Upper Gastrointestinal Lesions**

| **Disease progression stages** | **Utility score** | **Disease progression stages** | **Utility score** |
| --- | --- | --- | --- |
| Esophagus |  | Gastric |  |
| mD | 0.944 | CAG | 0.969 |
| MD | 0.939 | IM | 0.960^a^ |
| SD/CIS | 0.921 | LGIN | 0.930 |
| Early EC | 0.889 | HGIN | 0.922 |
| Advanced EC | 0.804 | Early GC | 0.828 |
| Cardia |  | Advanced GC | 0.773 |
| IM | 0.960^a^ |  |  |
| LGIN | 0.941 |  |  |
| HGIN | 0.927 |  |  |
| Early CC | 0.863 |  |  |
| Advanced CC | 0.724 |  |  |

Note：^a^Due to the small sample size of the field survey, it is obtained by reviewing the literature.

**3 Model validation**

The model-predicted life expectancy of the population was compared with the 2018 survey data from the Shandong center for disease control and prevention[21], and the results are shown in the **eTable11**. The model predicted a lower life expectancy than the actual survey results for people of all ages, ranging from -1.48 years to -2.24 years, which is perfectly acceptable[22]. Thus, the predictions were stable.

**eTable10. Comparison of model-predicted life expectancy with real data**

| **Age group** | **model predictions (year)** | **Survey data (year)** | **difference** |
| --- | --- | --- | --- |
| 40-44 | 38.12 | 40.36 | -2.24 |
| 45-49 | 33.42 | 35.58 | -2.16 |
| 50-54 | 28.93 | 30.87 | -1.94 |
| 55-59 | 24.57 | 26.28 | -1.71 |
| 60-64 | 20.36 | 21.89 | -1.53 |
| 65-69 | 16.35 | 17.83 | -1.48 |
| 70-74 | 12.67 | 14.19 | -1.52 |
| 75-79 | 9.45 | 11.14 | -1.69 |
| 80-84 | 6.67 | 8.62 | -1.95 |
| ≥85 | 4.70 | 6.44 | -1.74 |

**eResults**

**1 One-way sensitivity analysis**

**
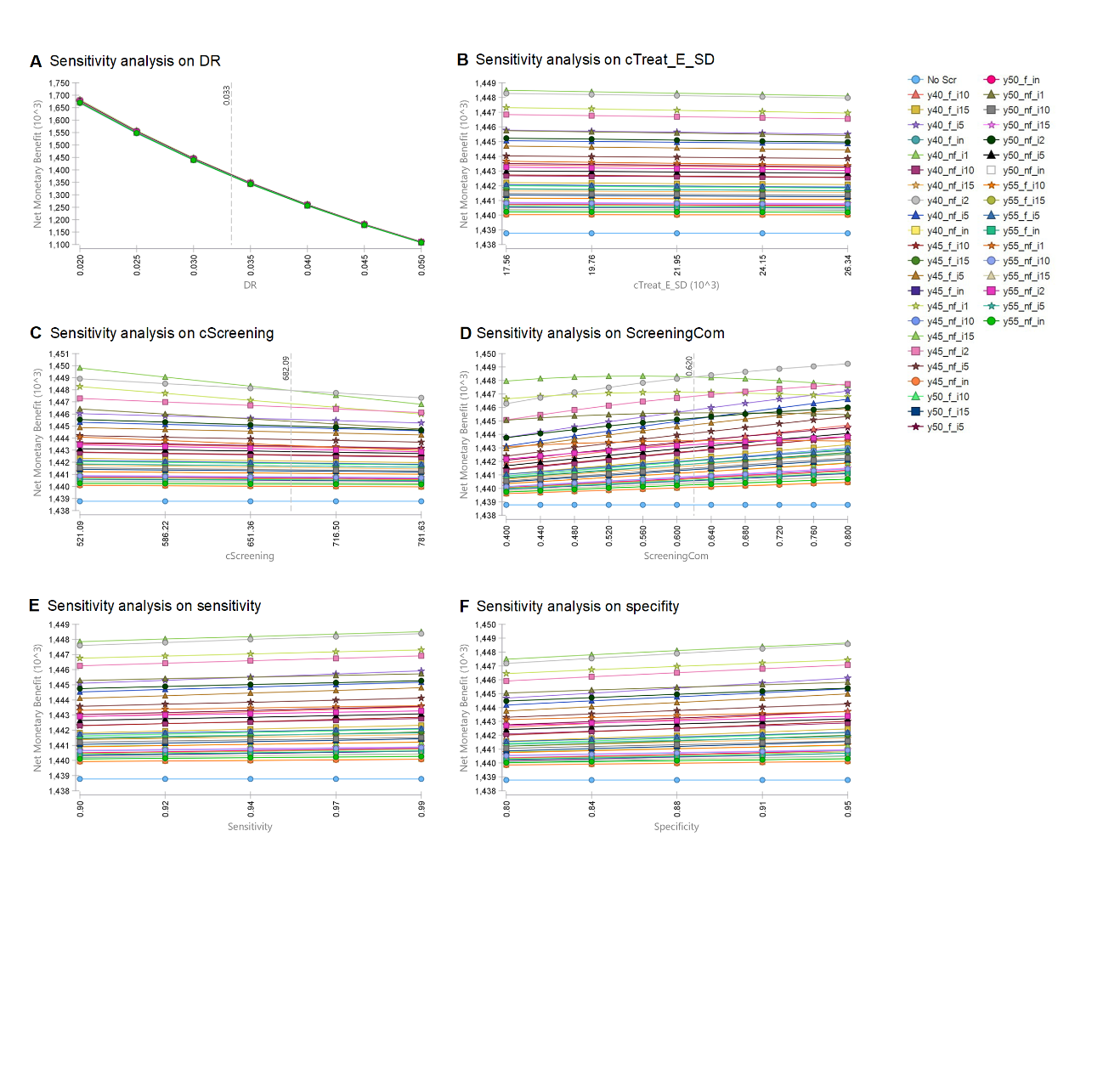
eFigure 2. One-way sensitivity analysis of primary factors**

Abbreviations: DR, discount rate; cTreat_E_SD: treatment cost for severe atypical hyperplasia; cScreening, screening cost; ScreeningCom, screening compliance.

**eReferene**

[1] National Clinical Research Center for Digestive Diseases (Shanghai), Chinese Society of Digestive Endoscopy Cancer, Chinese Endoscopist Association, Chinese Society of Digestive Endoscopy, Endoscopy Professional Committee of China Anti-Cancer Association.Expert consensus on the diagnosis and treatment strategy of pre-cancerous state and precancerous lesions of esophageal squamous carcinoma in China.Chinese Journal of Digestive Endoscopy, 2020,37(12):853-867.DOI: 10. 3760/cma.j.cn321463-20200928-00807.

[2] National Clinical Research Center for Digestive Disease (Shang hai), National Early Gastrointestinal Center Prevention & Treatment Center Alliance, Helicobacter Pylori Group, Chinese Society of Gastroenterology, Chinese Medical Association, et al. Chinese consensus on management of gastric epithelial precancerous conditions and lesions (2020) [J]. Chin J Dig, 2020 ,40 (11): 731-741. DOI:10. 3760/ cma.j.cn311367-20200915-00554.

[3] Correa P, Piazuelo MB. Helicobacter pylori Infection and Gastric Adenocarcinoma[J]. US Gastroenterol Hepatol Rev, 2011,7(1):59-64.

[4] Wang GQ, Wei WQ. Technical Program for Screening and Early Detection and Treatment of Upper Gastrointestinal Cancer (2020 Pilot Version) [M] ,2020.

[5] Yang J, Wei WQ, Niu J, et al. Cost-benefit analysis of esophageal cancer endoscopic screening in high-risk areas of China[J]. World J Gastroenterol, 2012,18(20):2493-2501. DOI: 10.3748/wjg.v18.i20.2493.

[6] Wen DG, Wang SJ, Zhang LW, Zhou W, Yu WF, Wang XL. Natural history of esophageal and gastric cardia precursor by repetitive endoscope screening with 425 adults in a high-risk area in China. Cancer Epidemiol 2009;33(2):108-12.

[7] Yang J, Wei WQ, Niu J, Liu ZC, Yang CX, Qiao YL. Cost-benefit analysis of esophageal cancer endoscopic screening in high-risk areas of China. World J Gastroenterol 2012;18(20):2493-501.

[8] Ma SR. Distributive Characteristics of Precancerous Lesions of Gastric Cardia Adenocarcinoma and its Progress Rate in High-risk Areas of Upper GI Cancer in China--- Population Based Muti-center Cohort Follow-up Study[D] : Peking Union Medical College,2017.

[9] Correa P, Haenszel W, Cuello C, Zavala D, Fontham E, Zarama G, Tannenbaum S, Collazos T, Ruiz B. Gastric precancerous process in a high risk population: cohort follow-up. Cancer Res 1990;50(15):4737-40.

[10] Zhao JM, Wang LZ, Wang HJ, Zhang AW, Wang HX. A follow-up outcome of 102 patients with chronic atrophic gastritis caused by HP infection for 10 years[J]. Modern Preventive Medicine. 2012. 39(15): 3958-3960.

[11] Zhang B, Linghu EQ, Chai NL, et al. Risk factors of recurrence for gastric mucosal lesions after endoscopic submucosal dissection[J]. Chinese Journal of Digestive Endoscopy. 2018. 35(01): 32-36.

[12] Liu M, Wang Y. Long-term follow-up in cardia intestinal metaplasia[J]. International Journal of Digestive Diseases. 2001. (02): 119.

[13] Ge JR, Dong LH, Chen LG, Zhang QY, Mao YL. Pathological changes of gastric mucosa after eradication of Helicobacter pylori [J]. Clinical Focus. 2005. (16): 911-913.

17. Zhang LW, Wang SJ, Yu WF, et al. The Value of Endoscopic Minor-injury Treatment for Early Cardia Cancer and Precancerous Lesion[A],2011.

[14] Kim JK, Kim GH, Lee BE, Park CH, Jeon HK, Baek DH, Song GA. Endoscopic submucosal dissection for esophagogastric junction tumors: a single-center experience. Surg Endosc 2018;32(2):760-9.

[15] Lai SQ, Wang GQ. Public Screening for Early Carcinoma of Gastric Cardia: Rule of Carcinogenetic Development Observed by Endoscopy. [J]. The Chinese-German Journal of Clinical Oncology,2006(02):93-95+148.

[16] Dixon MF. Gastrointestinal epithelial neoplasia: Vienna revisited. Gut 2002;51(1):130-1.

[17] Raftopoulos SC, Kumarasinghe P, de Boer B, Iacobelli J, Kontorinis N, Fermoyle S, Olynyk J, Forrest C, Ee HC, Yusoff IF. Gastric intraepithelial neoplasia in a Western population. Eur J Gastroenterol Hepatol 2012;24(1):48-54.

[18] Lin J. The study of the clinicopathological and endoscopic characteristics of early gastric cancer and precancerous lesions[D]: Zhejiang University,2015.

[19] Ma M, Xiao H, Li L, Yin X, Zhou H, Quan H, Ouyang Y, Huang G, Li X, Xiao H. Development and validation of a prognostic nomogram for predicting early recurrence after curative resection of stage II/III gastric cancer. World J Surg Oncol 2019;17(1):223.

[20] Zhou T, Ma AX. The survival analysis applied in calculation of Markov model transition probability in pharmaceutical evaluation[J]. Chinese Journal of Evidence-Based Medicine,2018,18(10):1129-1134.

[21] Lu ZL, Fu ZT, Du EQ, et al.A study on self-reported health-adjusted life expectancy of adults in Shandong Province，2018[J]. Journal of Shandong University (Health Sciences) ,2020,58(09):83-88. DOI: 10．6040 /j．issn．1671-7554．0．2020．0118.

[22] Wang J, Lei HC. Study on the Characteristics of Gaps Between Cohort and Period Life Expectancy[J]. Chinese Health Economics,2009,28(07):20-21.
